# About the Essence of Trust: Tell the Truth and Let Me Choose—I Might Trust You

**DOI:** 10.3389/ijph.2022.1604592

**Published:** 2022-05-25

**Authors:** Felix Gille

**Affiliations:** Digital Society Initiative, University of Zurich, Zurich, Switzerland

**Keywords:** concept, health care, trust, theory, trustworthiness

## Abstract

Trust is critical to the success of health care. We witness a diversification of trust constructs which is due to the context specificity of trust and the popularity of trust in marketing, public, and political debate. This diverse use of trust leads to confusion. To contribute to the dissolution of this confusion, I propose that the essence of trust follows Tell the truth and let me choose—I might trust you*.* Inferring from empirical and theoretical work, I argue that the conceptual framework of trust is comprised of: communication, truthful information, autonomy, alternatives, and no guarantee. This oversimplification aims to stimulate a debate about the common denominator of existing conceptual frameworks of trust in health care. By refining and agreeing on the core of trust in health care, we will be able to improve debate, improve comparability among trust studies, and improve understandability of policy recommendations to foster trust in health care.

## Preliminary

What reads as a bold simplification is my proposition of the essence of how to establish trust in medicine and health care. *Tell the truth and let me choose—I might trust you* is a concise answer to the question: how do you establish trust? The phrase condenses complex conceptual work to the key elements of building a trusting relationship between two parties: A freely chooses to trust B in anticipation of a benefit. A might choose to trust B based on truthful information communicated by B. As a result of this trusting relationship, A participates in health care activities and B’s action are legitimized by A. Both results are central to successful health care system governance, public health interventions, medical care, and recovery from illness. Truthful information and the freedom to place trust are the sticking points of all trust relationships. Following Niklas Luhmann, without free choice we are not able to place trust [1]; and following our personal experiences we tend to not trust someone or something that we consider untruthful.

## What Is the Problem?

The interest in trust and how to increase trust skyrocketed in recent years. This is not only visible within medicine and public health, but also in other areas of our life triggered by, for example, the last financial crisis, the European migration crisis, and the separation of states from established partnerships—Brexit. These are events that introduce uncertainty into a familiar and for many of us as stable perceived society and state system. The term trust is very prominent in the public sphere, for better or for worse. Yet, the prominence of the term trust in the public sphere might indeed hint towards the need to discuss issues of trust.

Simultaneously, trust research gained momentum to better understand how trust develops and what actors in health care systems can do to increase or maintain high levels of trust. It appears that the health care system is a particularly well researched area with respect to trust relationships, at the same time the health care system is a social and political area that enjoys considerable high degrees of trust in comparison to other areas such as the media or police. Hence, I argue that it is of value to locate this discussion in health care as an example for other social, political, and private systems that exist within societies. Standing on the shoulders of established trust theories [1–3], present trust research in health care focuses for example on adherence to COVID-19 measures; data sharing; health reforms; vaccination uptake, personalised medicine or the fight against misinformation and conspiracy theories.

As trust is an inherently complex construct and highly context specific, the research output makes a lot of sense when reading individual studies but can be confusing when comparing studies across the field. This problem has not changed for the better in years, I argue that it has worsened. This is so, as we see the emergence of “How to be trustworthy and to establish trust” guidelines in different health care areas that are not necessarily research based. One example is several guidelines for the introduction of artificial intelligence [4]. The combination of established knowledge on trust, the increasing research-based contemporary evidence on trust relationships in health care systems, and the range of not research-based practice guides on how to maintain and increase trust paints a Jackson Pollock style painting of what trust in health care is.

On top of everything, we use a single concept (literally the same word - trust) in a variety of health care contexts with very different contextual requirements towards trust. We understand trust to be important in the context of emergency care, purchase of insurances, doctor-patient relationships, vaccination, organ donation, data donation, and so forth. To illustrate two settings, in acute emergency care we might have little time to build trust, hence we might build trust based on recognizable labels, sleek workwear, and the knowledge of institutional and health system guarantees such as professional education or standard operating procedures. When participating in biobank research, we use the same word trust to describe the relationship between a donor and the institution, but the construct will be built based on entirely different contextual conditions. First, we have time to decide, we might have several consultations before we agree to participate and donate our samples. We can gather information from different sources, we have time to build relationships with biobank representatives and eventually we can also decide not to engage in the activity without personal direct consequences. We could separate the examples by arguing the first hinges on institutional guarantees, whereas the second example showcases how trust is built by personal relationships with health system representatives. Indeed, it is likely that on a system level trust is built by both. Yet, both examples show that when using trust in different health care settings, the underlying conceptual framework needs to be either very broad and abstract to cover a range of contexts or highly dynamic to adapt to the setting it is used in. How can a single construct work everywhere?

For us researchers, working with this conceptual muddle in large can be an entertaining and indeed knowledge rich activity with promising outputs. From a health professional, health policy maker, or politician perspective this puzzling picture is of little use—probably the only true main message is “It is confusing” or “It depends on the context.” Certainly, researchers usually translate findings into actionable policy guidelines, yet again, when comparing such guidelines, the picture remains somewhat abstract. This conceptual confusion weakens the health policy discourse. To make efficient health policy and health care planning, a clear understanding of the concept in focus is imperative [5].

Therefore, I propose the introductory paragraph as a response to the complexity of current trust research and the wish to provide the most simplistic explanation of what health care actors need to do to establish trust. What might read as reductionistic and certainly highly disputable is the quest to walk the opposite way of complex concept development. This is indeed an exercise to stimulate debate among health care users, trust researchers, health policy makers, and health care professionals about the essence of trust relationships in health care. Beyond the academic dispute, I anticipate that finding the common denominator of what trust in medicine and health care is has implications such as:

• Abstractification*—*If we can define a common understanding where we start and come back to, it will be much easier to walk up and down levels of abstraction within conceptual frameworks.
• Comparability*—*even when comparing studies in the same health care setting, we find authors using different conceptual frameworks despite claiming to study the same construct. This makes comparison of results and synthesis very difficult, if not impossible.
• Understanding*—*this might read trivial but given the complexity of trust it would be very useful for the discourse to have a common understanding of what we talk about when we talk about trust. Otherwise, it might be more like us discussing temperature where some of us follow Lord Kelvin, others Daniel Fahrenheit, and the remaining follow Anders Celsius.
• Transferability*—*with an agreed conceptual core, we can more easily (identify) what conceptual items are context specific and therefore better transfer concepts from one setting to the other.

## What Is the Essence of Trust in Health Care?

The proposed simplification *Tell the truth and let me choose—I might trust you* encompasses the quintessence of trust: communication, truthful information, autonomy, alternatives, no guarantee.

### Communication—Tell

Communication is the lifeblood of trust. By communicating truthful information, communication contributes to the establishment of trust and maintenance of a trusting relationship. Communication encompasses 1) active communication as for example during the medical consultation or the consent process; 2) passive communication via information campaigns and the media image of health care system actors; and 3) following signalling theory, signs and signals such as professional uniforms or respected labels communicate information necessary to build trust. I argue further that personal experiences and collective memory communicate direct or comparable information about the to be trusted other. For example, collective memories of the Tuskegee Syphilis Experiments (conducted from 1932 to 1972) in the African American community to this day foster mistrust towards health providers [6].

### Truthful Information—The Truth

The purpose of trust breeding communication is to convey truthful information [7]. The content of the truthful information will be in part context specific and represent the conceptual themes that make an actor trustworthy and lead eventually to the establishment of trust. For example, for public trust in health care activities, this information will relate to past positive experiences (e.g., familiarity with vaccinations and the positive experienced knowledge that the previous vaccination shots did not harm), present perceptions (e.g., presence of regulatory frameworks that can serve as assurances for the to be trusted action, the competences and skills of the to be trusted to achieve what s/he should be trusted for, or safety mechanism and quality control) and future anticipations (e.g., expected personal or collective benefit, for example that a vaccination will protect me and others from a disease) [8]. The question of what truth is and the ability of the to-be-trusting party to assess truth is indeed critical for the establishment of trust [9]. Within the doctor-patient relationship the knowledge gradient between health professionals and patients and contradicting professional opinions or facts; and in the public space, fake news, and misinformation as well as conspiracy theories are among the main obstacles which need to be overcome to be able to place trust. Con men, deception, and intentional communication of untruthful information are poison for trust building. Here, increased health literacy and communication strategies in easy language are key as well as equipping all of us with the skills necessary to distinguish truth from untruth [10]. Adherence to professional codes of conduct, morality and virtue, as well as professional ethics certainly work against the communication of untruth, but there will never be a guarantee that information is true. A risk of betrayal will remain in every trust relationship. Within a doctor-patient relationship, situations exist where truth telling for the doctor is difficult as for example when a patient does not want to hear about test results and therefore is not able to make an informed decision about future treatment. This example places additional value on autonomy, as introduced below. With autonomy, the to be trusting party can control how much truth they are willing to accept without placing the professional into compromising situations.

Following this plea for truth, you might ask why I advocate for truth and not for other commonly accepted themes of trust such as honesty, quality, or safety [11]. Honesty relates to a behavioural trait of health care actors to tell the truth and is often used almost as a synonym for or at least in the same breath as truth. Quality usually refers to high quality of care and safety to keep the patient safe during medical procedures or to keep the public safe for example from environmental health threats. I argue that quality and safety are highly important themes for trust but are not properties of information. Information about degrees of quality and safety need to be truthful to be useful for the establishment of trust. Honesty could indeed replace truth. However, I argue that honesty is closer related to transparency [12]. There is no doubt that to disentangle the concepts of truth and honesty is indeed a challenge and can be discussed in great length.

### Autonomy—Let Me

Misztal, 1996, argues that trust can only develop in a free society based on free will [13]. Also, on a personal level and within a free society, it is difficult to imagine how trust can be forced or expected, especially when, following Hartmann, 1994, the trustworthiness of a person can only be judged by others and not by the person herself [14]. An expectation to be trusted might develop from arrogance, hubris, thoughtlessness or from a long-exercised routine leading to a normative expectation. Closely linked to granted freedom, autonomy is a central element of decision making within health care and needs no further introduction. It seems very disturbing to imagine that trust should be established based on misuse of power. Certainly, within the health care system in some situations patients cannot exercise their autonomy, as for example when patients lack capacity to make decisions. This highlights the importance of ethical guidance and the presence of professionals and relatives to make decisions according to the will of the patient. The existence of regulatory frameworks, professional conduct and trusted intermediaries indeed fosters trust and can in these situations complement autonomy.

### Alternatives—Choose

To grant freedom in the health care system and to exercise autonomy, choices need to be offered and made. At minimum the choice to take part or not to take part in health care activities. Trust theory greatly centres around choices made in trust relationships and the importance of the ability to choose between alternatives. This focus on choice is especially relevant in behavioural economics research which uses for example game theory to research trust. Niklas Luhmann even goes as far as arguing that if there is no choice offered there is no place for trust [1]. You might challenge this existential position of choice with concepts such as generalized trust in the world or trust in God as a guardian over health care activities. Yet, I argue, the latter should actually be substituted with faith and the former relates to spontaneous sociability which is the ability to interact with unknown persons in an established set of norms and values. Both are related but distinct concepts of trust in health care.

### No Guarantee—I Might

So far, I have not seen any research that demonstrates a guaranteed way to establish trust. A lot of research provides excellent guidance striving to trust establishment, yet there is no guarantee. I mentioned above that an expectation to be trusted is foolish. Further, present trust research tends to conceptualize trust as if trust is built by calculated decisions. As if we have a checklist in our head and when all boxes are ticked, we trust. Maybe this is true for some, for others emotion and feeling are the trigger to place trust [15]. Sometimes it is a very diffuse vague feeling that is difficult to describe. Some of us might say “it just feels right to trust.” We can agree that a health care system actor can do a lot of things right that certainly contribute to the establishment of trust, but there will never be a guarantee. We need to live with this uncertainty.

### Conclusion

I propose what I consider to be the essence of the concept of trust in health care and medicine: truth and freedom. This exercise comes with caveats as discussed above but can also be beneficial if understood as the common denominator of the many trust conceptualizations that exist. Such highly condensed conceptual understanding can serve as a common starting ground for comparative research, can be translated in easy-to-understand policy guidelines and is highly transferable across settings. Now, I invite others to debate and contribute to the refinement of the proposed simplification with the aim to establish a commonly agreed and accepted understanding of what trust in health care is.

## Data Availability

The original contributions presented in the study are included in the article/supplementary material, further inquiries can be directed to the corresponding author.
